# 

**Published:** 1888-05-05

**Authors:** 


CONTENTS.

**Buried Alive!** - 69

**Words of Consolation.**—LXXV. - 70

**The Doctor's Pulpit.**—Constitution Building - 71

**American Jottings** - 72

**Scotch Physicians and Surgeons.** By C. G. FURLEY.—James Syme - 73

**Physiology and its Uses.**—Bones and Backbones - 74

**Notes and News** - 76

**Everybody's Page** - 78

**Annotations.**—Will Workmen Give?—Can Workmen Pay?—Pasteurism and Hydrophobia - 79

**An Ishmaelite.** By LESLIE KEITH, Author of "The Chilcotes," "East and West," etc. - 80

**Patients' Contributions to Hospitals.** By W. BURDETT-COUTTS, M.P. - 81

**Healthy Hospitals** - 83

**Amusements with Profit for all Ages.**—For Men and Women.—Children's Corner.—Puzzles, etc. - 84

**EXTRA SUPPLEMENT.—THE NURSING MIRROR.**

En Passant.—A Hard Case.—Directory for Nurses.—Cottage Nurses.—The Pay of Private Nurses.—Metropolitan Nursing Association.—Convalescent Homes.—The Queen's Nursing Fund.—Female Skulls.—Poultices.—*Work and Leisure* - xiii
The National Pension Fund for Nurses.—The Case of the Older Nurses—The National Pension Fund and the British Nurses' Association—Applicants' Difficulties - xiv
Everybody's Opinion—Anecdotes of Personal Adventure—Appointments—Vacancies.—Notes and Queries.—Exchange and Mart - xv
Lectures—Examination Questions - xvi

